# ST3Gal.I sialyltransferase relevance in bladder cancer tissues and cell lines

**DOI:** 10.1186/1471-2407-9-357

**Published:** 2009-10-07

**Authors:** Paula A Videira, Manuela Correia, Nadia Malagolini, Hélio J Crespo, Dário Ligeiro, Fernando M Calais, Helder Trindade, Fabio Dall'Olio

**Affiliations:** 1 CEDOC, Departamento de Imunologia, Faculdade de Ciências Médicas, FCM, Universidade Nova de Lisboa, Lisboa, Portugal; 2 Department of Experimental Pathology, University of Bologna, Bologna, Italy; 3 Centro de Histocompatibilidade do Sul, Lisboa, Portugal; 4 Grupo Português Génito-Urinário, Hospital São José, Lisboa, Portugal

## Abstract

**Background:**

The T antigen is a tumor-associated structure whose sialylated form (the sialyl-T antigen) involves the altered expression of sialyltransferases and has been related with worse prognosis. Since little or no information is available on this subject, we investigated the regulation of the sialyltransferases, able to sialylate the T antigen, in bladder cancer progression.

**Methods:**

Matched samples of urothelium and tumor tissue, and four bladder cancer cell lines were screened for: *ST3Gal.I*, *ST3Gal.II *and *ST3Gal.IV *mRNA level by real-time PCR. Sialyl-T antigen was detected by dot blot and flow cytometry using peanut lectin. Sialyltransferase activity was measured against the T antigen in the cell lines.

**Results:**

In nonmuscle-invasive bladder cancers, *ST3Gal.I *mRNA levels were significantly higher than corresponding urothelium (p < 0.001) and this increase was twice more pronounced in cancers with tendency for recurrence. In muscle-invasive cancers and matching urothelium, *ST3Gal.I *mRNA levels were as elevated as nonmuscle-invasive cancers. Both non-malignant bladder tumors and corresponding urothelium showed *ST3Gal.I *mRNA levels lower than all the other specimen groups. A good correlation was observed in bladder cancer cell lines between the *ST3Gal.I *mRNA level, the ST activity (r = 0.99; p = 0.001) and sialyl-T antigen expression, demonstrating that sialylation of T antigen is attributable to ST3Gal.I. The expression of sialyl-T antigens was found in patients' bladder tumors and urothelium, although without a marked relationship with mRNA level. The two *ST3Gal.I *transcript variants were also equally expressed, independently of cell phenotype or malignancy.

**Conclusion:**

ST3Gal.I plays the major role in the sialylation of the T antigen in bladder cancer. The overexpression of *ST3Gal.I *seems to be part of the initial oncogenic transformation of bladder and can be considered when predicting cancer progression and recurrence.

## Background

Bladder cancer is one of the most common cancers in humans and its incidence has been increasing during the past years [1]. 70-80% of all bladder cancers are nonmuscle-invasive and have a low mortality rate. However, despite the complete resection of the primary lesion, and the success of treatments such as the intravesical instillation with bacillus Calmette-Guérin (BCG) [2], 30-50% of patients with nonmuscle invasive bladder cancer experience recurrence within the first year after resection and 15% of these patients manifest worsening of tumor grade and stage. For those reasons, bladder cancers are becoming a serious public health problem and a social and economic burden.

The oligosaccharide chains of glycoproteins and glycolipids are often decorated by sialic acids, a family of nine carbon sugars derived from neuraminic acid. In humans, sialylation of glycoconjugates is mediated by different sialyltransferase enzymes which, depending on their nature, may establish different types of linkages [linkage through an α2-3- or an α2-6-bond to galactose (Gal); through an α2-6-bond to N-acetylgalactosamine (GalNAc) or N-acetylglucosamine (GlcNAc); or through an α2-8-bond to another sialic acid, forming polysialic acid] (reviewed in [3]). During neoplastic transformation, the activity of sialyltransferases may be altered and, as a consequence, cancer cells express more heavily sialylated glycans at the surface [4]. This aberrant sialylation may mediate key pathophysiological events during the various steps of tumor progression, including invasion and metastasis formation. This is due to the fact that sialylated structures can prevent cell-cell interactions through non-specific charge repulsion, but they can be specifically bound by cell adhesion molecules, such as selectins [5]. On the other hand, the addition of sialic acids may mask the underlying sugar structure, thus avoiding recognition by other specific glycan binding molecules, such as galectins [6]. Moreover, specific sialylated structures, aberrantly expressed in carcinomas, have been used as targets for cancer immunotherapy in preclinical and clinical studies [7-9].

The T antigen, or Thomsen-Friedenreich antigen, is a simple glycan, formed by the dissacharide, galactose (Gal) β1-3-linked to N-acetylgalactosamine (GalNAc), O-glycosidicaly-linked to serine or threonine [10]. The addition of sialic acid to this core 1 structure, forming the sialyl-T antigen, inhibits any further chain extension (with the exception of the possible addition of another sialic acid to GalNAc). These antigens are largely increased in some types of cancers (colon, prostate, cervix, ovary, breast) and, according to the above mentioned role of sialylation, the sialylated form (sialyl-T) has been associated with worse prognosis (reviewed in [11]). According to the known specificities, three sialyltransferases, namely ST3Gal.I, ST3Gal.II and ST3Gal.IV can mediate the sialylation of the T antigen and the increase in the expression of these sialyltransferases has been shown to be one of the major mechanisms responsible for the sialylation of T antigen [10]. There is growing evidence that these molecules can act as good markers in cancer. In fact, in breast cancer, ST3Gal.I has been found increased compared with normal tissue and its expression is related to the grade of the tumor [12]. Altered mRNA expressions of these sialyltransferases were also shown to be of importance in malignant epithelial ovarian cancers [13] and in colon carcinoma [14,15].

The relevance of sugar structures related with the T antigen in the control of bladder cell proliferation is indicated by the fact that the antiproliferative factor (AFP), a sialoglycopeptide comprised of the sialyl-T trisaccharide linked to a peptide derived from a membrane receptor [16], which is secreted by bladder cells of interstial cystitis patients, exerts a strong inhibitory effect on bladder cell proliferation, both *in vivo *and *in vitro*. Nevertheless, in spite of the few studies dealing with the expression of T and sialyl-T antigens [17-19] or other types of sialylated structures [20,21] in bladder cancer and there are no published data about the expression of related sialyltransferases.

In this study, we attempted to investigate the relevance of the sialyltransferases ST3Gal.I, ST3Gal.II or ST3Gal.IV expression in bladder cancer progression, by comparing their expression in neoplastic tissue, from different grade bladder tumors, with those of normal urothelium. We found that the mRNA levels of *ST3Gal.I *were significantly higher in malignant bladder tumors. The analysis of four bladder cancer cell lines showed that this sialyltransferase gives the most relevant contribution for the sialylation of T antigens in bladder cancer cells.

## Methods

### Patient and tissue specimens

This study involves 54 patients [mean age of 65.3 years (range 45 to 80)], who underwent bladder surgery, between July 2005 and July 2007, due to various indications. Matched pairs of histologically verified bladder tumors and normal appearing mucosa remote from the tumor were collected from patients with diagnosed nonmuscle-invasive bladder cancer (Group I, n = 43) or invasive bladder cancer (Group II, n = 6). Patients with carcinoma in situ (CIS) and cancers with low risk for progression and recurrence (primary TaG1 bladder cancers) were excluded from this study. To serve as control, 4 patients with benign tumors (papilloma) were included (Group III). Within the group of patients with nonmuscle-invasive bladder cancer, further subgroup was studied: Group IA-patients with no recurrence or progression in a minimum period of 12 months (n = 29) and Group IB-patients with subsequent recurrence or progression in less than 12 months (n = 14). Both subgroups received the same treatment: transurethral resection of the bladder tumors (TURBT) followed by the same dose and schedule of BCG instillations (TICE^® ^BCG). A summary of the clinical data is available in Table 1. Patients with at least one of the following criteria were excluded from this study, i.e., presence of upper tract malignancy, other malignancies, chronic infections, women expectant or lactating and congenital or acquired immunodeficiency. For the use of these clinical materials, prior patient consent and approval from the institute research ethics committee were obtained.

**Table 1:** Stage and grade distribution of bladder cancer in our study patients

**Patients groups and subgroups**^a^	**I**^b^**(n = 43)**	II (n = 6)
		
	IA (n = 29)/IB (n = 14)	
	**Number of cases per group**	

**Stage**		

Ta	18 (41.9%)	-
		
	14 (48.3%)/4 (28.5%)	

T1	25 (58.1%)	-
		
	15 (51.7%)/10 (71.4%)	

T2	-	6 (100%)

**Stage/grade**		

TaG1^c^	12 (27.9%)	-
		
	9 (31.0%)/4 (28.5%)	

TaG2	6 (13.9%)	-
		
	5 (17.2%)/-(0%)	

T1G1	6 (13.9%)	
		
	5 (17.2%)/1 (7.1%)	

T1G2	14 (32.6%)	
		
	7 (24.1%)/7 (50.0%)	

T1G3	5 (11.6%)	
		
	3 (10.3%)/2 (14.3%)	

T2G2	-	3 (50%)

T2G3	-	3 (50%)

^a^The control group III used in this study was omitted from the table since it does not includes malignant tumors.

^b^The group II of patients were all treated by TURBT followed by the same dose and schedule of BCG instillations (TICE^® ^BCG).

^c^Patients with low risk, primary TaG1 cancers were not included in the study.

After collection, samples were immediately divided: a fraction was immersed in RNAlater^® ^RNA Stabilization Reagent (Sigma Aldrich Inc., St. Louis, MO, USA) and then preserved at -20°C and another fraction was stored at -80°C, until further processing.

### RNA isolation and characterization

Samples preserved in RNAlater (20 to 50 mg) were homogenized and the total RNA was isolated using the GenElute Mammalian Total RNA Purification kit and DNAase treatment (Sigma), according to the manufacturer's instructions. RNA concentrations were measured and only samples with A_260_/A_280_ratios between 1.9 and 2.1 were considered further. 250 to 500 ng of total RNA (1 μg for cell lines) was reverse transcribed into cDNA, by using the random-primers-based High Capacity cDNA Archive Kit (Applied Biosystems, Foster City, CA, USA).

### Gene expression measurements

Reverse transcriptase-polymerase chain reaction (RT-PCR) in real time was performed using Taqman probes methodology, mainly as we described [22,23]. For each primer/probe set, the Assay ID (Applied Biosystems), the detected reference sequences and the location on the gene were the following: *ST3Gal.I *(Hs00161688_m1; NM_173344.2NM_003033.3; exons 5, 6), *ST3Gal.II *(Hs00199480_m1; NM_006927.2; exons 1, 2) and *ST3Gal.IV *(Hs00272170_m1; NM_006278.1; exons 1, 2). The mRNA expression was normalized using the geometric mean of the expression of the endogenous controls, *β-actin *and *GAPDH *genes [23]. The relative mRNA levels were calculated by using the adapted formula 2^-ΔCt ^*1000[24], which infers the number of mRNA molecules of a certain gene per 1000 molecules of the endogenous controls. ΔCt stands for the difference between the cycle threshold of the target gene and that of the endogenous control genes. The efficiency of the amplification reaction for each primer-probe was above 95% (determined by the manufacturer).

### Cell lines

The human bladder cancer cell lines, HT1374, MCR, T24 and 5637, were grown in Dulbecco's modified Eagle medium (DMEM) (Sigma), supplemented with 10% foetal calf serum (Sigma), 2 mM L-glutamine and 100 μg/ml penicillin/streptomycin.

### Sialyltransferase assay

Sialyltransferase activity was assayed in whole cell homogenates as we previously described [25,26], using Galβ1,3GalNAcα1-O-benzyl (Sigma), as acceptor. The enzyme reaction was performed at 37°C for 2 hours and then the products were isolated by hydrophobic chromatography, eluted and counted by liquid scintillation. Controls without exogenous acceptors were run in parallel and their incorporation was subtracted. The activity was measured as the amount of radioactive sialic acid transferred from the donor to the acceptor substrate, per hour and per amount of protein (nmol/h* μg protein).

### Neuraminidase treatment

Cells were resuspended in DMEM medium (5 × 10^6^/ml) and treated with 200 mU/ml neuraminidase from *Clostridium perfringens *(Roche Diagnostics GmbH, Mannheim, Germany) in serum-free medium at 37°C for 90 min. In the case of dot blots, 25 μg of protein homogenate were treated with 0.8 mU/μl neuraminidase at 37°C for 6 hours. In parallel, identical samples were mock-treated in the same conditions with heat-inactivated neuraminidase (neuraminidase previously submitted 15 min at 100°C).

### Analysis of the phenotypic expression of T antigen by flow cytometry

Staining with *Arachis hypogaea *lectin, a peanut agglutinin (PNA) was used to examine the expression of T antigens in the cell lines. 10^5 ^cells, treated or not with neuraminidase, were resuspended in serum free RPMI medium and incubated with 50 μg/ml of Fluorescein isothiocyanate (FITC)-labelled PNA (EYLaboratories, San Mateo, CA), for 15 min, at 18°C in the dark. Cells were then washed and resuspended in FACS Flow supplemented with 0.1% BSA. Cells were then analyzed in a FacsCalibur Flow cytometer using CellQuest software (Becton Dickinson), following standard procedures.

### Lectin blot analysis

The expression of T antigens in tissue samples was assessed by PNA lectin dot blot. 12 μg of protein from neuraminidase treated and mock-treated samples were transferred to a Hybond nitrocellulose membrane (GE Healthcare). After blocking, the membranes were stained with PNA-digoxigenin conjugate (1 μg/ml) for 1 h and subsequently incubated with 7.5 U/μl anti-digoxigenin antibody conjugated with horse-radish peroxidase (Roche). After washing, the membranes were incubated with Immobilon™ Western Chemiluminescent Substrate (Millipore) according to manufacturer's instructions and detected with autoradiography film.

### Analysis of *ST3Gal.I *transcript variants

Comparison between *ST3Gal.I *mRNA (Reference Sequences: NM_003033.2 and NM_173344.2) and genomic sequences [NCBI http://www.ncbi.nlm.nih.gov and Ensembl http://www.ensembl.org databases] were performed using Basic Local Alignment Search Tool (BLAST) and refined manually. In order to estimate the size of *ST3Gal.I *transcript and distinguish its two variants in bladder cancer cells, 2 μl of cDNA (obtained as referred above) were amplified by conventional RT-PCR, using forward primers located in exon Y (F1: 5'-CTATGCCAGACAGTTTCGACA-3'), exon ×1 (F2: 5'-CAAGAGCTGCAGTGAGCAAA-3') and exon 1 (F3: 5'-CGACAGGATGGGAAAGAAAA-3'); and reverse primers located in exon 1 (R1: 5'-TGTGGGAGTAGTTCAGGAAGAA-3'), exon 2 (R2: 5'-CTCCAGCATAGGGTCCACAT-3') and exon 6 (R3: 5'-AGACATGCTCTGCCACGC-3'). PCR amplification was carried out with a 5 min of preincubation at 95°C, followed by 35 cycles of the following profile: denaturation at 95°C for 60 s, annealing at 55°C for 60 s, and extension at 72°C for 60 s. PCR products were analyzed on a 2% agarose gel stained with ethidium bromide. In some cases their identity was confirmed by DNA sequencing.

### Statistical analysis

Data from healthy urothelium were paired with data from tumor tissue and statistically analyzed using Kruskal-Wallis (one-way ANOVA) test. The correlation between the sialyltransferase gene expression and activity, in the cell lines, was analyzed using Spearman and Pearson methods. All statistical tests were considered statistically significant when p < 0.05. All data are expressed as the mean ± SEM. Analyses were conducted using GraphPad Prism software, version 5.0 (GraphPad Software, La Jolla, CA).

## Results

### *ST3Gal.I *expression is increased in bladder cancer

**Figure 1:**
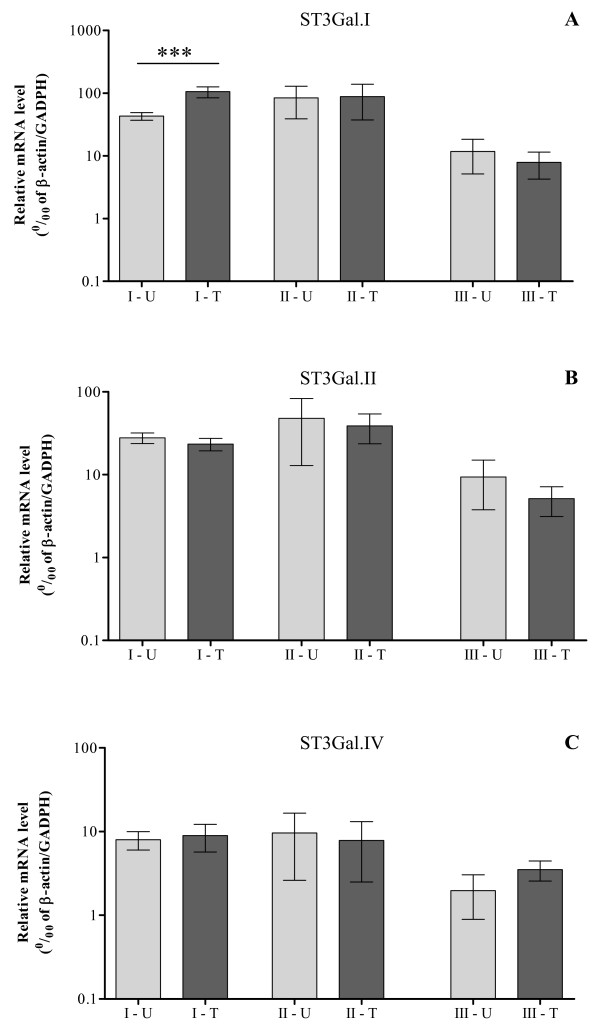
**Bladder cancer tissue has increased expression of *ST3Gal.I***. The relative mRNA levels of *ST3Gal.I *(**A**), *ST3Gal.II *(**B**) and *ST3Gal.IV *(**C**) genes were analyzed by Real Time RT-PCR as described in the Methods section, in bladder tumor tissue (T) and corresponding normal urothelium (U) from patients with nonmuscle-invasive (I), with invasive (II) and with non malignant bladder tumors (III). Values infer the number of mRNA molecules of a certain gene per 1000 molecules of the average of the endogenous controls (β-actin and GAPDH). *ST3Gal.I *expression is significantly increased in tissue of nonmuscle-invasive bladder cancer (I), compared with the corresponding urothelium (p < 0.001).

To understand whether there is an altered expression of ST3Gal.I, ST3Gal.II or ST3Gal.IV in bladder tumors, we analyzed their mRNA levels. In the three analyzed groups of patients, we found that *ST3Gal.I *expression was significantly increased in tissue of nonmuscle-invasive bladder cancer (group I), compared with corresponding urothelium [105.3 ± 20.1 (tumors) and 43.0 ± 6.1 (urothelium) relative mRNA levels; p < 0.001] (Fig 1). In tissues from invasive bladder cancer (group II) and from non malignant bladder tumors (group III), *ST3Gal.I *expression was not significantly different from the corresponding urothelium. Interestingly, in group II of patients, *ST3Gal.I *transcripts ranged from 84.1 ± 32.2 (urothelium) to 88.0 ± 37.2 (tumors) mean relative mRNA levels, and were approximately eight times higher than group III, which ranged from 11.7 ± 6.6 (urothelium) to 7.9 ± 3.6 (tumors) mean relative mRNA levels (Fig 1A).

The expression of *ST3Gal.II *was not statistically different between tumor tissue and normal urothelium in all the analyzed groups of patients. However, in the group II of patients, *ST3Gal.II *mean mRNA levels were twice higher [ranging from 47.9 ± 20.9 (urothelium) to 38.8 ± 11.2 (tumors)] than in group I [ranging from 27.8 ± 4.1 (urothelium) to 23.4 ± 4.0 (tumors)] and five times higher than group III [ranging from 9.4 ± 3.6 (urothelium) to 5.1 ± 2.0 (tumors)] (Fig 1B).

Similarly, while *ST3Gal.IV *expression was also not statistically different between tumor tissue and normal urothelium in all the analyzed groups of patients, in samples from patients with non malignant tumors (group III) its mRNA expression was 3 to 4 times lower [ranging from 1.9 ± 1.1 (urothelium) to 3.5 ± 0.9 (tumors)] than samples from other patient's groups. Taken as a whole, the *ST3Gal.IV *mRNA levels were quite inferior to the mRNA levels of the *ST3Gal.I *and *ST3Gal.II *(Fig 1C).

Although, not statistically significant, we observed unexpected differences mRNA sialyltransferase expression levels among the normal urothelium of patients from the three groups. This is most evident for ST3Gal.I, which is expressed at the lowest level by patients who developed non malignant tumors (group III), at an intermediate level by those who developed malignant but nonmuscle-invasive cancers (group I) and at the highest level by those who developed invasive cancers (group II). These data can be interpreted as the result of a "field effect" of the tumor, whose presence and degree of malignancy would affect sialyltransferase expression also in apparently uninvolved tissues [27].

### Nonmuscle-invasive bladder cancer with tendency for recurrence show pronounced *ST3Gal.I *mRNA increase and decreased *ST3Gal.II *mRNA levels

33% of the patients with nonmuscle-invasive bladder cancer, included in this study, have experienced recurrence in less than 12 months after TURBT plus intravesical BCG. To elucidate whether the expression of *ST3Gal.I*, *ST3Gal.II *or *ST3Gal.IV *genes was dissimilar among patients without recurrence (Group IA) and those with subsequent recurrence (Group IB), we examined the transcripts levels of these sialyltransferases in these subgroups of patients.

**Figure 2:**
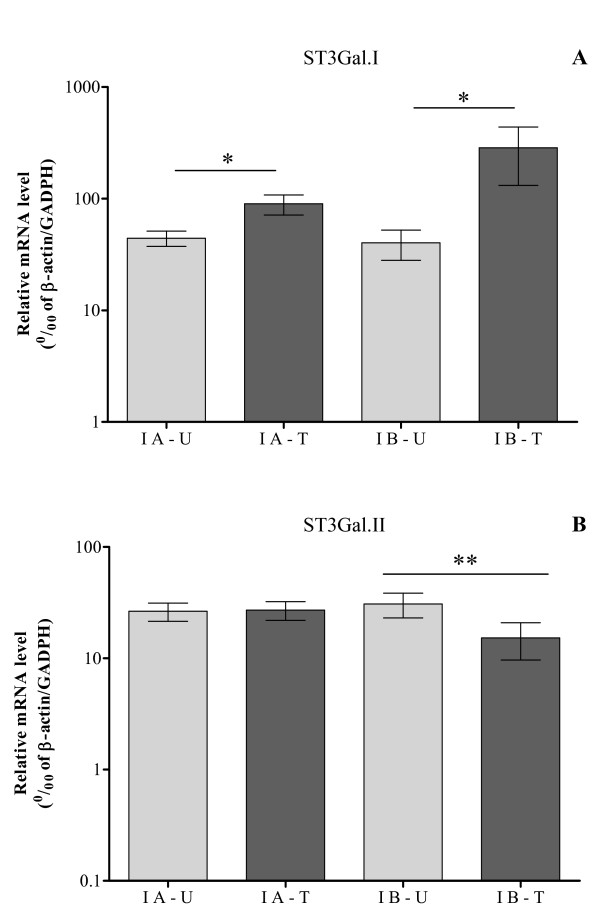
**Nonmuscle-invasive bladder cancers with tendency for recurrence show slight increased expression of *ST3Gal I *and *ST3Gal.II*, compared with normal urothelium**. Only patients with nonmuscle-invasive bladder cancer were considered: without recurrence within a minimal period of 12 months following TURBT (IA) and experiencing recurrence in that period (IB). The relative mRNA levels of *ST3Gal.I *(**A**) and *ST3Gal.II *(**B**) genes were analyzed in bladder tumor (T) tissue and corresponding normal urothelium (U), by Real Time RT-PCR, as described in the Methods section. Values infer the number of mRNA molecules of a certain gene per 1000 molecules of the average of the endogenous controls (β-actin and GAPDH). *ST3Gal.I *expression is significantly increased in tissue of both subgroups of nonmuscle-invasive bladder cancer, compared with the corresponding urothelium (p < 0.05), while *ST3Gal.II *expression is significantly decreased in tumors from group IB patients, compared with the corresponding urothelium (p < 0.01).

Compared to urothelium, *ST3Gal.I *mRNA levels were significantly higher in both tumors from group IA and group IB (p < 0.05) (Fig 2A). However, *ST3Gal.I *expression was remarkably higher in tumors from group IB of patients (285.3 ± 93.6) than tumors from group IA (89.8 ± 18.3) (p = 0.058) (Fig 2A), suggesting that a high level of *ST3Gal.I *expression is associated with propensity to recurrence. Conversely, *ST3Gal.II *mRNA expression, in group IB of patients, was significantly decreased in tumors (15.3 ± 5.6), compared to urothelium (30.7 ± 7.6) (p = 0.0046) (Fig 2B). In group IA, no significant differences were found for the expression of this gene. *ST3Gal.IV *mRNA levels were not significantly different among group IA and group IB of patients (not shown). All together, these data show that patients with nonmuscle-invasive bladder cancer, with tendency for recurrence present a significant augment in *ST3Gal.I *and reduction in *ST3Gal.II *expression in their tumors.

### ST3Gal.I is responsible for the sialylation of T antigens in bladder cancer cell lines

**Figure 3:**
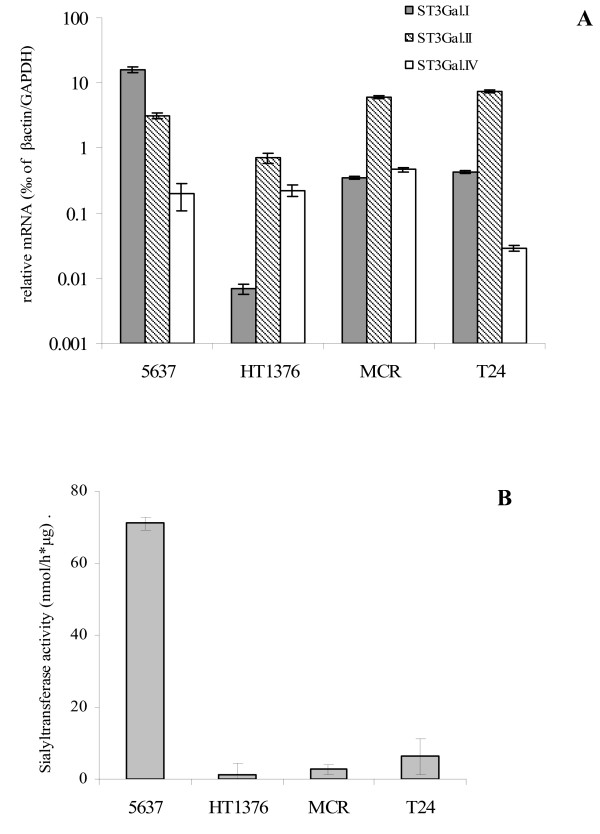
**The *ST3Gal.I *expression correlates with the sialyltransferase activity towards T antigen in bladder cancer cell lines**. **A**: The relative mRNA levels of *ST3Gal.I*, *ST3Gal.II *and *ST3Gal.IV *genes were analyzed in 5637, HT1376, MCR and T24 bladder cancer cell lines, by Real Time PCR. Values infer the number of mRNA molecules of a certain gene per 1000 molecules of the average of the endogenous controls (β-actin and GAPDH). Results are the mean of 3 independent assays. **B**: The sialyltransferase activity towards Galβ1,3GalNAcα1-O-Benzyl glycoside was assessed in sample homogenates of 5637, HT1376, MCR and T24 bladder cancer cell lines, as described in the Methods section. In the four analyzed bladder cancer cell lines, the *ST3Gal.1 *mRNA levels are correlated with the observed sialyltransferase activity towards the T antigen (r = 0.99; p = 0.001).

To investigate whether, in bladder cancer, there was a correlation between the activity of at least one of the ST3Gal.I, ST3Gal.II or ST3Gal.IV sialyltransferases and the expression of sialyl-T antigens, we examined in detail four distinct bladder cancer cell lines. According to the transcriptional analysis, the *ST3Gal.I *gene gave the most scattering expression among the four cell lines used in this study (Fig 3A). The 5637 cell line showed the highest expression for *ST3Gal.I *(16.0 ± 1.8 relative mRNA levels); contrasting with the HT1376 cell line, which showed the smallest expression and the MCR and T24 cell lines which expressed approximately forty times less *ST3Gal.I *mRNA levels than the 5637 cell line (Fig 3A). *ST3Gal.II *transcript levels were higher in the MCR and T24 cell lines (6.1 ± 0.4 and 7.5 ± 0.4, respectively), followed by the 5637 cell line, which showed around half expression, and the HT1376 cell line which showed one tenth of this expression (Fig 3A). The *ST3Gal.IV *gene was poorly expressed in all the four cell lines, ranging from 0.03 ± 0.003 to 0.46 ± 0.022 relative mRNA molecules (Fig 3A).

**Figure 4:**
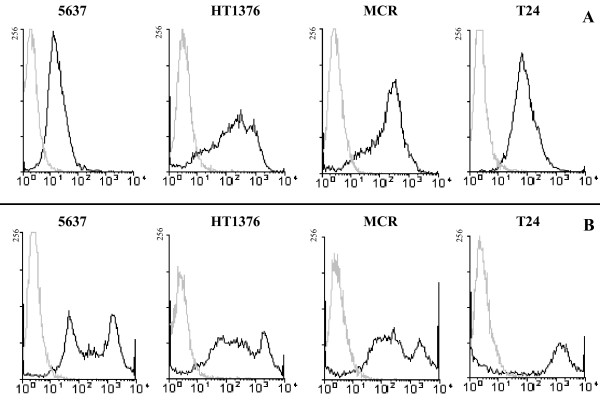
**Bladder cancer cell lines express both T and sialyl-T antigens**. The bladder cancer cell lines, 5637, HT1376, MCR and T24, treated with heat-inactivated neuraminidase (**A**) or with active neuraminidase (**B**) were analyzed by Flow cytometry using fluorescent PNA lectin. The gray line shows the controls without PNA. The T antigen is expressed at high levels by the HT1376 and MCR cell lines, but only weakly by 5637 cells and at an intermediate level by T24. According to the results obtained after removing the cell surface sialic acid by neuraminidase treatment, the 5637 and T24 cell lines express the T antigen masked by sialic acid.

Regarding the analysis of sialyltransferase activity, we observed that only 5637 cells show a high level of activity towards T antigens while, in the other three cell lines, the activity was very low or undetectable (Fig 3B). Among the analyzed genes, the expression of *ST3Gal.I *gave the best correlation with the observed sialyltransferase activity towards the T antigen (r = 0.99; p = 0.001). According to the cell staining with PNA, a lectin which recognizes the T antigen [28], it was evident that the T antigen was expressed at high levels by the HT1376 and MCR cell lines, but only weakly by 5637 cells and at an intermediate level by T24 (Fig 4). After removing the cell surface sialic acid, by neuraminidase treatment, part of the 5637 and T24 become PNA positive, indicating that these cell lines express the T antigen masked by sialic acid (Fig 4). These data propose the ST3Gal.I sialyltransferase is the major responsible for the sialylation of T antigens in bladder cancer cells.

### Sialyl-T antigens are expressed in bladder tumor tissue and urothelium

**Figure 5:**
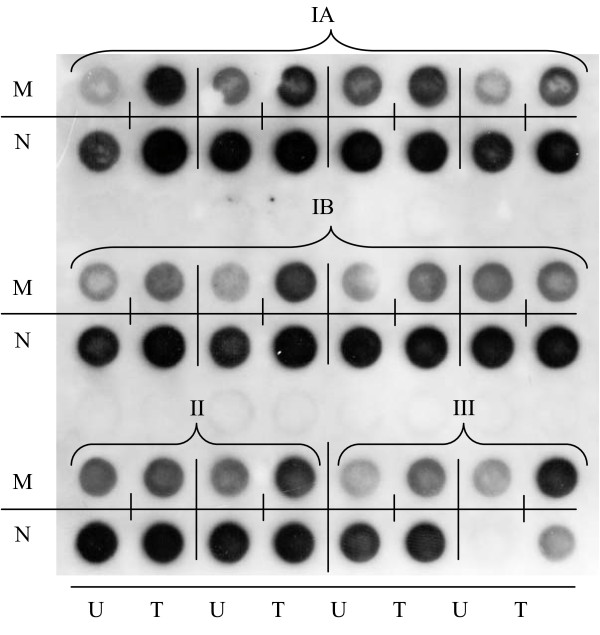
**T and sialyl-T antigens are expressed in bladder tumor tissue and urothelium**. Tumor (T) and Urothelium (U) samples from patients were analyzed by dot blot staining with PNA, which binds to T-antigen. Samples were arranged by tumor type, namely, nonmuscle-invasive without (IA) and with recurrence (IB) and muscle-invasive (II) bladder cancer and non-malignant tumors (III) and by treatment with neuraminidase (N) or mock treated (M). PNA binds to non neuraminidase treated samples, of all patients' groups, indicating the presence of T-antigens. In the majority of the samples, PNA binding is strongly increased after neuraminidase treatment.

To ascertain whether sialyl-T antigens were also expressed in the normal urothelium and bladder tumor specimens, we performed a PNA dot blot analysis. The majority of the analyzed samples stained with PNA (Fig 5), demonstrating the expression of T antigen. It appears that tumor samples had enhanced reactivity with PNA, but we couldn't find any peculiarity among the tumor malignancies and grades. After neuraminidase treatment, the PNA staining increased in nearly all the samples (Fig 5), demonstrating that part of the T antigens is masked by sialic acid, in the urothelium and bladder tumors. Altogether, these results suggest that tumors may have slightly increased expression of T antigens compared with urothelium, but we failed to find a significant different expression of T and sialyl-T antigens, between the different grades of tumors.

### Bladder cancer cells express two *ST3Gal.I *transcript variants

In the first paper describing the molecular cloning of human ST3Gal.I [29], it was reported the presence of two types of *ST3Gal.I *cDNAs which differ in the 5'untranslated region; and the shorter of the two transcript variants misses one or more exons. While the sequences of these two *ST3Gal.I *transcript variants were deposited at GenBank (Reference Sequences: NM_003033.2 and NM_173344.2 for the longer and shorter form, respectively), there was no further reference in the literature concerning this issue. In addition, it was not consensual whether there were six or seven exons in the coding region.

**Figure 6:**
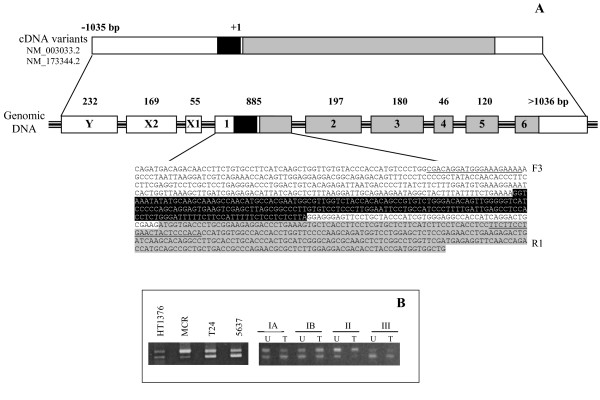
**The two *ST3Gal.I *transcripts are expressed in the majority of the bladder cancer and urothelium cells**. **A**: Schematic diagram of human *ST3Gal.I *cDNA (on the top) and genomic (on the centre) sequences. The black rectangle in *ST3Gal.I *cDNA represents the sequence which is present in *ST3Gal.I *transcript variant NM_003033.2 and absent on the transcript variant NM_173344.2. Exons containing the coding region (represented in grey) are denoted as 1-6 with the sizes shown above. The total of exon 1 nucleotide sequence is shown below: DNA sequence absent in transcript variant NM_173344.2 is represented on black background and the location of the primers F3 and R1 used to discriminate the transcript variants is underlined. **B**: RT-PCR analysis of NM_003033.2 and NM_173344.2*ST3Gal.I *transcripts in bladder cancer cell lines and patient's specimens. The *ST3Gal.I *exon 1 was amplified in cDNAs obtained from HT1374, MCR, T24 and 5637 bladder cancer cell lines and bladder tumor (T) tissue and corresponding normal urothelium (U) from 6 randomly chosen patients (subgrouped as described above), using F3 and R1 primers. The predicted sizes of the products are 609 bp (NM_003033.2) and 406 bp (NM_173344.2).

In a first investigation, we compared the two transcript variants and the genomic *ST3Gal.I *sequences. According to our *in silico *analysis, the shorter *ST3Gal.I *cDNA (NM_173344.2) does not lack any specific exon at the 5'untranslated region, but a 203 nucleotide sequence, within exon 1, 51 nucleotides upstream the translation initiation codon (Fig 6A). Both transcript variants are composed of nine exons with six of them containing the coding region (Fig 6A). The PCR amplification of cDNAs from the bladder cancer cell lines, using primers located in different *ST3Gal.I *exons, originated products whose sizes were in agreement with the *in silico *analysis and therefore presuppose the existence of two *ST3Gal.I *transcripts, each with nine exons (data not shown). The 203 nucleotides shortage which distinguish NM_173344.2 from NM_003033.2 transcript was confirmed by cDNA amplification, using primers located at the extremities of exon 1 (Fig 6A), where two PCR products with the expected size (609 bp and 406 bp) were obtained (Fig 6B), and by sequencing in both directions.

Although the intensities of the PCR products differed among the analyzed cell lines, all samples expressed both transcripts (Fig 6B). The two *ST3Gal.I *transcripts were also observed in the patient's specimens, with no significant differences regarding their nature (urothelium or tumor tissue) and the tumor grade (Fig 6B).

Considering the augmented *ST3Gal.I *mRNA expression which we observed in bladder cancer, it was not possible to correlate it with one of the transcripts. Although further studies should be conducted, these results suggest that, in these bladder cancer tissue and urothelium, both *ST3Gal.I *transcripts are randomly expressed, with no particular association with the overall expression of the gene and the type of tissue.

It is relevant to note that during the *in silico *analysis we have observed that the shorter cDNA had the highest identity (89%) with a pseudogene located at chromosome 4, followed by homology with a chimpanzee pseudogene, also chromosome 4, and then by genomic *ST3Gal.I *sequence, at chromosome 8. This sequence has no homology with any viral or bacterial gene and it is not flanked by any insertion element, which excludes the hypothesis of a pathogenic chromosome insertion.

## Discussion

A great part of the aberrant glycosylation found in cancer cells is due to the altered expression of sialyltransferases. These enzymes are considered as prognostic factors in some cancers [11] and recent data has revealed that they are important targets for therapeutic approaches [30,31]. Investigating the relevance of sialyltransferases in cancer is however a complex task, due the diversity expressed by human cells and for presenting some overlapping substrate specificities, tissue-restricted patterns of expression and individual variations.

Our aim was to investigate, in bladder cancers, the expression of the sialyltransferases, ST3Gal.I, ST3Gal.II and ST3Gal.IV, which are proposed to have a decisive role in the T antigen sialylation [10]. Our study with different groups of patients with bladder tumors, demonstrate markedly that the *ST3Gal.I *mRNA levels increases during the malignant transformation of urothelium. In fact, malignant bladder tumors show increased *ST3Gal.I *mRNA levels when compared with non malignant tumors. Particularly, tumors from patients with nonmuscle-invasive bladder cancers show significant increased *ST3Gal.I *mRNA levels compared to normal urothelium and, in patients where cancer penetrates the muscle layer (muscle-invasive), the increased *ST3Gal.I *mRNA is also observed in the apparent normal mucosa. This unexpected observation can be explained by at least two non mutually exclusive mechanisms. First, it can be hypothesized that the tumor exerts a kind of "field effect" which through the release of soluble mediators or by other means alters the basic sialyltransferases expression level in normal tissues. Interestingly, this effect is positively related with malignancy, since the level of *ST3Gal.I *in urothelium is the lowest in patients bearing non malignant tumors, intermediate in the group with nonmuscle-invasive cancer and highest in patients with invasive cancer. Second (but less likely), it is possible that individuals with a high basic level of *ST3Gal.I *in urothelium, are more prone to the development of invasive bladder cancers.

Interestingly, when further analysing specimens from patients with nonmuscle-invasive bladder cancer, subgrouped according to the development of recurrence, we found that tumors of patients who experienced subsequent recurrence showed higher increased expression of *ST3Gal.I *and decreased expression of *ST3Gal.II *(Fig 2). Therefore, our findings suggest that alterations in *ST3Gal.I *and *ST3Gal.II *transcript levels may anticipate recurrence propensity. Nevertheless, since we could not obtain higher number of patients within the defined inclusion criteria, the relevance of these data should be further substantiated. Even so, to our knowledge, this is the first report demonstrating that, likewise to other cancers types [13,15], the bladder cancer tissue also presents a deregulated expression of sialyltransferases.

Through the study of different bladder cancer cell lines it was possible to establish a functional correlation between ST3Gal.I and the manifestation of sialyl-T antigens at cell surface. Nevertheless, in patient specimens, it was not possible to confirm this correlation. Surprisingly, bladder tumor tissues, independently of their malignancy or grade, show higher PNA reactivity than the corresponding urothelium, suggesting the expression of higher content of non sialylated T antigens. One hypothesis to explain this discrepancy between *ST3Gal.I *expression and PNA reactivity is the fact that, in bladder cancer tissue, the ST3Gal.I is acting specifically on a limited number of molecules and any resulting increased content of sialyl-T containing glycans, due to its activity, is not perceptible by the adopted methodology, the dot blot analysis. Another possibility is that in cancer tissues there is a strong expression of T antigen molecules which cannot be fully sialylated neither in the presence of increased ST3Gal.I activity. Thirdly, there may exist translational regulation mechanisms of ST3Gal.I or the competition with other glycosyltransferase, such as core 2 β1-6 GlcNAc transferases, which compete with ST3Gal.I for core 1, generating the core 2 structures instead of sialyl-T antigens [10].

For this study, we selected the staining with PNA lectin which recognizes non sialylated T antigens and the expression of sialyl-T antigens is inferred after desialylating the tissue homogenates with neuraminidase treatment. This staining method is largely used for the detection of T and sialyl-T antigens in human normal and malignant tissues because it requires little amount of samples [32,33]. The reactivity of PNA lectin with bladder cancer specimens has been already studied by other authors [34,35], but the conclusions were controversial: while some [34] only observed PNA reactivity after neuraminidase treatment of bladder tumors, others [35] observed positive reactivity of PNA lectin in tumors without neuraminidase treatment, which was roughly consistent with the results here presented.

Interestingly, in a great part of other cancers, where a deregulated sialyltransferase expression is observed, the enzyme/product relationship is not direct (reviewed in [11]). Also, the use of cell line experimental models to correlate the overexpression of a given sialyltransferase and cancer-associated phenotypes provide results sometimes conflicting, mostly because of the differences between the glycosidic acceptors of each tumor type and each cell line [36].

*ST3Gal.I *gene is known to code for two transcript variants encoding the same protein [29]. It was suggested that a longer *ST3Gal.I *transcript could play an important role in the translational control of the expression of ST3Gal.I, since its longest 5' untranslated region contains multiple upstream ATG codons and open reading frames ("minicistrons") which could repress translation [29]. In this study, we observed that both transcripts were expressed in the majority of all the analyzed samples, both in healthy urothelium and tumor tissue, suggesting that the differential expression of these transcripts is not dictated by the malignant transformation of the cells. Apparently, the two transcripts were expressed in bladder cancer cells expressing different levels of *ST3Gal.I *mRNA and having different levels of ST3Gal.I activities. Albeit further functional investigations should be addressed, we suggest that, in bladder cancer, both transcripts have a redundant role and none of them have a particular role in the translational control of the expression of ST3Gal.I.

While we revised the characteristics of *ST3Gal.I *transcripts, two interesting features were observed: 1) the fact that the shorter *ST3Gal.I *transcript lacks a sequence within exon 1, at the 5' untranslated region, and 2) the identification of a pseudogene with high homology with *ST3Gal.I *cDNA. Similar chromosome insertion of "cDNA" was not found in any other sialyltransferase member and its origin is not explicable by the present genomic paradigms. Probably, some gene transcripts were processed by internal or external reverse transcriptases and inserted in genome during evolution. Nevertheless, it remains to clarify whether this sequence plays any role in regulating *ST3Gal.I *expression.

## Conclusion

Collectively, the data presented in this paper supports the hypothesis that aberrant expression of the sialyltransferases is part of initial oncogenic transformation of bladder. ST3Gal.I plays the major role in the T antigen sialylation, and its expression is associated with bladder cancer malignancy and recurrence. However, further work is required to identify the involved molecular events and assess the ST3Gal.I role, aiming the identification of potential immunotherapeutic targets. Nevertheless, our findings suggest that the sialyltransferase mRNA levels, in patient's bladder tissue, are parameters to be considered when predicting progression and recurrence of cancer.

## List of abbreviations used

FITC: Fluorescein isothiocyanate; Gal: galactose; GalNAc: N-acetylgalactosamine; PNA: peanut agglutinin; PCR: polymerase chain reaction; RT-PCR: Reverse transcriptase-polymerase chain reaction.

## Competing interests

The authors declare that they have no competing interests.

## Authors' contributions

PAV and FD designed the study, interpreted the results and wrote the manuscript. MC and PAV carried out all the gene expression assays and data organization. NM and FD analyzed the activity and phenotype of bladder cancer cell lines. HJC performed dot blot analysis and drew the graphics. FMC gathered patient biopsies and clinico-pathological data. HT contributed by evaluating the study and conceding financial support. DL and PAV performed the variant transcript analysis. All authors approved the final manuscript.

## Pre-publication history

The pre-publication history for this paper can be accessed here:

http://www.biomedcentral.com/1471-2407/9/357/prepub

## References

[B1] Lamm DL McGee WR Hale K Bladder cancer: current optimal intravesical treatment Urol Nurs 2005 25 323 332 16294610

[B2] Huben RP Intravesical chemotherapy versus immunotherapy for superficial bladder cancer Semin Urol Oncol 1996 14 1 Suppl 1 17 22 8727806

[B3] Harduin-Lepers A Vallejo-Ruiz V Krzewinski-Recchi MA Samyn-Petit B Julien S Delannoy P The human sialyltransferase family Biochimie 2001 83 727 737 10.1016/S0300-9084(01)01301-3 11530204

[B4] Hakomori S Glycosylation defining cancer malignancy: new wine in an old bottle Proc Natl Acad Sci USA 2002 99 10231 10233 10.1073/pnas.172380699 12149519 PMC124893

[B5] Dimitroff CJ Pera P Dall'Olio F Matta KL Chandrasekaran EV Lau JT Bernacki RJ Cell surface N-acetylneuraminic acid alpha2,3-galactoside-dependent intercellular adhesion of human colon cancer cells Biochem Biophys Res Commun 1999 256 631 636 10.1006/bbrc.1999.0388 10080950

[B6] Fuster MM Esko JD The sweet and sour of cancer: glycans as novel therapeutic targets Nat Rev Cancer 2005 5 526 542 10.1038/nrc1649 16069816

[B7] Holmberg LA Oparin DV Gooley T Sandmaier BM The role of cancer vaccines following autologous stem cell rescue in breast and ovarian cancer patients: experience with the STn-KLH vaccine (Theratope) Clin Breast Cancer 2003 3 Suppl 4 S144 51 10.3816/CBC.2003.s.004 12620152

[B8] Chefalo P Pan Y Nagy N Guo Z Harding CV Efficient metabolic engineering of GM3 on tumor cells by N-phenylacetyl-D-mannosamine Biochemistry 2006 45 3733 3739 10.1021/bi052161r 16533056 PMC2531244

[B9] O'Boyle KP Coatsworth S Anthony G Ramirez M Greenwald E Kaleya R Steinberg JJ Dutcher JP Wiernik PH Effects of desialylation of ovine submaxillary gland mucin (OSM) on humoral and cellular immune responses to Tn and sialylated Tn Cancer Immun 2006 6 5 16524255

[B10] Brockhausen I Pathways of O-glycan biosynthesis in cancer cells Biochim Biophys Acta 1999 1473 67 95 10580130 10.1016/s0304-4165(99)00170-1

[B11] Dall'Olio F Chiricolo M Sialyltransferases in cancer Glycoconj J 2001 18 841 850 10.1023/A:1022288022969 12820717

[B12] Burchell J Poulsom R Hanby A Whitehouse C Cooper L Clausen H Miles D Taylor-Papadimitriou J An alpha2,3 sialyltransferase (ST3Gal I) is elevated in primary breast carcinomas Glycobiology 1999 9 1307 1311 10.1093/glycob/9.12.1307 10561455

[B13] Wang PH Lee WL Juang CM Yang YH Lo WH Lai CR Hsieh SL Yuan CC Altered mRNA expressions of sialyltransferases in ovarian cancers Gynecol Oncol 2005 99 631 639 10.1016/j.ygyno.2005.07.016 16112178

[B14] Kudo T Ikehara Y Togayachi A Morozumi K Watanabe M Nakamura M Nishihara S Narimatsu H Up-regulation of a set of glycosyltransferase genes in human colorectal cancer Lab Invest 1998 78 797 811 9690558

[B15] Schneider F Kemmner W Haensch W Franke G Gretschel S Karsten U Schlag PM Overexpression of sialyltransferase CMP-sialic acid:Galbeta1, 3GalNAc-R alpha6-Sialyltransferase is related to poor patient survival in human colorectal carcinomas Cancer Res 2001 61 4605 4611 11389097

[B16] Keay SK Szekely Z Conrads TP Veenstra TD Barchi JJ Jr Zhang CO Koch KR Michejda CJ An antiproliferative factor from interstitial cystitis patients is a frizzled 8 protein-related sialoglycopeptide Proc Natl Acad Sci USA 2004 101 11803 11808 10.1073/pnas.0404509101 15282374 PMC511055

[B17] Langkilde NC Wolf H Clausen H Kjeldsen T Orntoft TF Nuclear volume and expression of T-antigen, sialosyl-Tn-antigen, and Tn-antigen in carcinoma of the human bladder. Relation to tumor recurrence and progression Cancer 1992 69 219 227 10.1002/1097-0142(19920101)69:1<219::AID-CNCR2820690136>3.0.CO;2-A 1727666

[B18] Yokoyama M Ohoka H Oda H Oda T Utsumi S Takeuchi M Thomsen-Friedenreich antigen in bladder cancer tissues detected by monoclonal antibody Hinyokika Kiyo 1988 34 255 258 3376817

[B19] Yamada T Fukui I Yokokawa M Oshima H Changing expression of ABH blood group and cryptic T-antigens of noninvasive and superficially invasive papillary transitional cell carcinoma of the bladder from initial occurrence to malignant progression Cancer 1988 61 721 726 10.1002/1097-0142(19880215)61:4<721::AID-CNCR2820610415>3.0.CO;2-5 3338034

[B20] Przybylo M Hoja-Lukowicz D Litynska A Laidler P Different glycosylation of cadherins from human bladder non-malignant and cancer cell lines Cancer Cell Int 2002 2 6 10.1186/1475-2867-2-6 12234377 PMC140134

[B21] Kajiwara H Yasuda M Kumaki N Shibayama T Osamura Y Expression of carbohydrate antigens (SSEA-1, sialyl-Lewis X, DU-PAN-2 and CA19-9) and E-selectin in urothelial carcinoma of the renal pelvis, ureter, and urinary bladder Tokai J Exp Clin Med 2005 30 177 182 16285609

[B22] Videira PA Calais FM Correia M Ligeiro D Crespo HJ Calais F Trindade H Efficacy of Bacille Calmette-Guerin Immunotherapy Predicted by Expression of Antigen-presenting Molecules and Chemokines Urology 2009 74 4 944 50 10.1016/j.urology.2009.02.053 19428084

[B23] Videira PA Ligeiro D Correia M Trindade H Gene expression analysis in superficial bladder cancer: comparison of two suitable endogenous reference genes Curr Urol 2007 1 145 150 10.1159/000115377

[B24] Livak KJ Schmittgen TD Analysis of relative gene expression data using real-time quantitative PCR and the 2(-Delta Delta C(T)) Method Methods 2001 25 402 408 10.1006/meth.2001.1262 11846609

[B25] Dall'Olio F Malagolini N Guerrini S Lau JT Serafini-Cessi F Differentiation-dependent expression of human beta-galactoside alpha 2,6-sialyltransferase mRNA in colon carcinoma CaCo-2 cells Glycoconj J 1996 13 115 121 10.1007/BF01049687 8785482

[B26] Videira PA Amado IF Crespo HJ Alguero MC Dall'Olio F Cabral MG Trindade H Surface alpha 2-3- and alpha 2-6-sialylation of human monocytes and derived dendritic cells and its influence on endocytosis Glycoconj J 2008 25 259 268 10.1007/s10719-007-9092-6 18080182

[B27] Heney NM Daly J Prout GR Jr Nieh PT Heaney JA Trebeck NE Biopsy of apparently normal urothelium in patients with bladder carcinoma J Urol 1978 120 559 560 712898 10.1016/s0022-5347(17)57275-4

[B28] Pereira ME Kabat EA Lotan R Sharon N Immunochemical studies on the specificity of the peanut (Arachis hypogaea) agglutinin Carbohydr Res 1976 51 107 118 10.1016/S0008-6215(00)84040-9 1033785

[B29] Kitagawa H Paulson JC Differential expression of five sialyltransferase genes in human tissues J Biol Chem 1994 269 17872 17878 8027041

[B30] Hsu CC Lin TW Chang WW Wu CY Lo WH Wang PH Tsai YC Soyasaponin-I-modified invasive behavior of cancer by changing cell surface sialic acids Gynecol Oncol 2005 96 415 422 10.1016/j.ygyno.2004.10.010 15661230

[B31] Zhu Y Srivatana U Ullah A Gagneja H Berenson CS Lance P Suppression of a sialyltransferase by antisense DNA reduces invasiveness of human colon cancer cells in vitro Biochim Biophys Acta 2001 1536 148 160 11406350 10.1016/s0925-4439(01)00044-8

[B32] Amado M Yan Q Comelli EM Collins BE Paulson JC Peanut agglutinin high phenotype of activated CD8+ T cells results from de novo synthesis of CD45 glycans J Biol Chem 2004 279 36689 36697 10.1074/jbc.M405629200 15210702

[B33] Shio Y Suzuki H Kawaguchi T Ohsugi J Higuchi M Fujiu K Kanno R Ohishi A Gotoh M Carbohydrate status detecting by PNA is changeable through cancer prognosis from primary to metastatic nodal site: A possible prognostic factor in patient with node-positive lung adenocarcinoma Lung Cancer 2007 57 187 192 10.1016/j.lungcan.2007.02.007 17383051

[B34] Langkilde NC Orntoft TF A comparative study of peanut agglutinin and amaranthin binding to human urinary bladder tumor glycoproteins Scand J Urol Nephrol Suppl 1995 172 57 64 8578258

[B35] Nakanishi K Kawai T Suzuki M Lectin binding and expression of blood group-related antigens in carcinoma-in-situ and invasive carcinoma of urinary bladder Histopathology 1993 23 153 158 10.1111/j.1365-2559.1993.tb00473.x 8406387

[B36] Malagolini N Santini D Chiricolo M Dall'Olio F Biosynthesis and expression of the Sda and sialyl Lewis × antigens in normal and cancer colon Glycobiology 2007 17 688 697 10.1093/glycob/cwm040 17395692

